# Management of ocular surface disease involving inflammation and persistent epithelial defects utilising various treatment modalities in the UK National Health Service (NHS)

**DOI:** 10.1038/s41433-026-04314-6

**Published:** 2026-06-04

**Authors:** Sundas Ejaz Maqsood, John William Posnett, Mohamed Elalfy

**Affiliations:** 1 Corneo-Plastic Unit, Queen Victoria Hospital NHS, East Grinstead, UK; 2 Eye Department, Maidstone and Tunbridge Wells Hospitals, Maidstone, UK; 3 Cornea Department, Research Institute of Ophthalmology, Giza, Egypt; 4 Health Economist, York, UK

**Keywords:** Health care, Business and industry

## Abstract

**Objective:**

To evaluate clinical outcomes and resource utilisation of conventional treatment modalities for ocular surface disease involving inflammation and persistent epithelial defect (PED) in an NHS setting, and to compare outcomes with sutureless human amniotic membrane-derived dry matrix (HAMDM) as an alternative therapy.

**Methods:**

A retrospective clinical study of 30 patients with PED was conducted across two centres. Treatment duration, healing rates, outpatient (OP) burden, and associated costs were analysed. Outcomes were benchmarked against pooled data from prior studies with HAMDM at the same centres.

**Results:**

Conventional management achieved complete healing in 57% of patients, with a mean treatment duration of 176 ± 188 days and 10 OP visits per case. Adjunctive procedures such as tarsorrhaphy (83%) and amniotic membrane transplantation (AMT, 34%) were frequently required. The average total cost per patient was £ 4900 ± £ 3363. In comparison, patients treated with HAMDM achieved higher resolution rates (63%), shorter treatment duration (98 days), fewer OP appointments (8 per case), and a lower treatment cost.

**Conclusion:**

PED poses a substantial clinical and economic burden within the NHS. Standard treatments are prolonged and resource-intensive. HAMDM represents a clinically effective and cost-saving alternative, potentially reducing treatment duration, OP burden and overall healthcare costs. Hospital Episode Statistics (HES) data revealed over 138,000 PED-related episodes annually in the NHS, primarily managed in non-surgical settings. These findings therefore support further integration of sutureless amniotic membrane into first-line care strategies for ocular surface disease involving inflammation and PED.

## Introduction

Persistent corneal epithelial defects (PED, or PCED) are a serious ocular surface pathology that represents a clinical management challenge and a significant burden on healthcare systems. A healthy, intact corneal epithelium is essential for preserving vision and protecting the eye from external insults [1]. Disruptions to the epithelial barrier and basement membrane due to trauma, infection, surgery, or systemic disease can lead to non-healing defects, placing patients at risk of complications such as corneal melting, scarring, secondary infection, and eventual perforation[1].

A PED is typically defined by the failure of corneal re-epithelialisation beyond 10–14 days, despite appropriate therapeutic measures [2, 3]. This differentiates it from minor epithelial injuries, which normally resolve within 1–2 weeks [1]. In clinical practice, PEDs often become chronic, requiring long-term therapy and frequent follow-up, and may progress to more severe outcomes, including irreversible vision loss [2–4].

PEDSs arise from a complex interplay of exogenous and endogenous factors. These include ocular surface trauma (mechanical, chemical), neurotrophic conditions, microbial keratitis, limbal stem cell deficiency (LSCD), postoperative complications, and systemic autoimmune disease [2, 4–7].

Management of PED presents a clinical challenge, largely dependent on the underlying cause [4]. The diverse aetiology necessitates a multifactorial treatment approach that aims to protect the eye, address the underlying cause of the defect and foster an environment conducive to epithelial healing [2]. Treatment is typically escalated in a stepwise manner[8], beginning with intensive lubrication and progressing through adjunctive therapies such as bandage contact lenses (BCL), punctal plugs, tarsorrhaphy, immunosuppressives, serum eye drops, and surgical options like amniotic membrane transplantation (AMT) or limbal stem cell (LSC) grafting [1, 4, 5, 9–17].

Despite the clinical significance of PEDs, there remains a lack of real-world data on the treatment burden and cost of managing these cases within the UK NHS. Much of the existing literature focuses on individual modalities, specific causes, or case series from tertiary centres, with few studies exploring longitudinal treatment pathways or associated healthcare costs. The variability in management, the absence of unified coding for PED in national data systems, and the heterogeneity in clinical outcomes further complicate evaluation of service impact and economic efficiency [18, 19].

The National Health Service (NHS), like many public health systems, is increasingly strained by chronic, resource-intensive conditions. In ophthalmology, outpatient (OP) services have seen significant growth in demand, with corneal surface disorders among the top contributors to repeated visits and treatment complexity. Chronic PEDs, though relatively infrequent in incidence, generate a high cumulative burden due to prolonged healing time, frequent monitoring, and the need for surgical rescue therapy.

This study aimed to retrospectively assess the clinical outcomes, treatment burden, and direct economic costs associated with the management of PED at two major NHS hospitals. Through a detailed audit of real-world data, it sought to:

- Characterise the aetiology, treatment pathways, and clinical resolution rates of PED

- Quantify OP visit frequency, duration of care, and associated costs

- Compare standard treatment outcomes and costs with those from a previously published cohort using a sutureless human amniotic membrane-derived dry matrix (HAMDM).

By exploring both clinical and economic aspects of PED management, this study aims to provide evidence to support improved treatment strategies and ophthalmology service planning within the NHS.

## Methodology

### Study design and setting

A retrospective clinical study was conducted to evaluate treatment outcomes and resource utilisation associated with the management of ocular surface diseases, specifically PED, across two UK NHS centres: Queen Victoria Hospital Foundation Trust, East Grinstead, and Maidstone and Tunbridge Wells Trust, Kent. The study period spanned from 2017 to 2022. This project received local institutional approval at both hospitals and adhered to the tenets of the Declaration of Helsinki.

### Patient inclusion criteria

Eligible patients were those diagnosed with ocular surface disease in either acute and chronic (persistent) phases, presenting with PED, with or without associated ocular surface inflammation. PED was defined, in line with established criteria, as a failure to complete epithelialisation 10–14 days after onset or injury, despite appropriate standard therapeutic intervention [2, 20]. Only cases with clear documentation of a refractory healing course and escalation or management, indicating PED most difficult to treat, were included.

### Data extraction from patient records

Relevant clinical data were extracted from patient medical records, including demographics, diagnosis, treatment interventions, clinical outcomes, duration of treatment, number of outpatient (OP) visits, medications prescribed/treatments administered, and any additional procedures or follow-up treatments. Cost-related data were calculated based on these extracted values.

### Outcome measures

The primary outcomes assessed were: (i) clinical resolution status; (ii) treatment duration; (iii) number of OP appointments required; (iv) direct OP treatments and related costs; and (v) follow-up treatments. Clinical outcomes were categorised as complete healing (defined as total epithelial closure and resolution of inflammation), partial healing (defined as reduction in epithelial defect size or severity), no change, or deterioration over the treatment course. The number and frequency of OP attendances were recorded from the date of initial presentation to final documented resolution or discharge.

### Cost analysis

Cost calculations were based on NHS Reference Costs and British National Formulary (BNF) pricing available at the time of the study. The cost analysis aimed to capture the economic burden of managing patients with PED under standard-of-care (SOC) pathways. The following components were included in the cost model:

#### Outpatient (OP) appointment costs

The cost of the initial OP consultation was set at £134, based on 2017 NHS Reference Costs [21]. Each subsequent follow-up OP visit was costed at £59. The number of OP appointments per patient was recorded from clinical records and multiplied by the corresponding cost per visit. The total OP appointment cost per patient was therefore calculated using £134 for the initial assessment and then £59 for each of the follow-up visits.

#### Treatment cost per OP appointment

For each patient, the combination of conventional topical therapies (including lubricants, topical antibiotics, and corticosteroids) and treatments was recorded and using averaged BNF pricing, the assumption was that a uniform medication cost was applied at each OP visit until resolution. This standardised value was used as the ‘Prescribed treatment cost per OP visit’ and used to calculate the average cost across the full PED cohort, reflecting average prescribing practices observed in the audit.

#### ‘Total OP treatment cost’

This value represents the total cost incurred during OP treatment from initial presentation to clinical outcome (either resolution or final status). It includes the total OP appointment cost and the cumulative cost of medications and documented interventions:

#### Patient follow-up intervention costs

Some patients require further follow-up treatments, such as surgical intervention (e.g. corneal grafts, amniotic membrane transplantation, or stem cell transplantation), long-term treatments (e.g. serum eye drops or long-term BCL treatment), or long-term follow-up. The costs of these interventions were calculated based on NHS surgical tariffs or published unit costs.

For procedures without a fixed tariff, an average theatre cost of £20 per minute was applied based on national estimates. Unit costs for treatments like serum drops and immunosuppressants were derived from the BNF or published literature. Instances of non-attendance were also noted and factored into overall per-patient costs. These values are presented as the ‘Patient follow-up intervention cost’ and exclude additional OP visits or medication costs incurred during follow-up.

#### Overall (Final) cost per-patient

The total cost burden for each patient was calculated by combining the OP treatment cost with the follow-up treatment costs. This value reflects the complete cost from presentation through to clinical resolution or outcome.

### Comparison with human amniotic membrane-derived dry matrix (HAMDM)

Outcomes from the SOC cohort in this study were compared with pooled outcomes from two previously published clinical studies involving treatment with a sutureless human amniotic membrane-derived dry matrix (HAMDM; Omnigen® with OmniLenz, NuVision Biotherapies, UK) [1, 22]. Outcomes data from the two previous HAMDM studies were pooled and compared to data from this study of SOC (Table 1). Patients in the comparator group were treated with HAMDM as a primary or adjunctive intervention. Because all treatments occurred at the same NHS sites and under the same clinical teams, it was assumed that treatment pathways and institutional protocols were comparable.

**Table 1:** Cost comparison of standard of care (SOC) vs. pooled data of HAMDM [1, 22] for refractory PED.

	SOC (This study)	HAMDM (Maqsood) [1, 22]	% Saving with AMDDM
*Total Follow-up time (days)*	176.0	98.1	
*OP appointments*	10.13	8.00	
Complete Resolution (%)	57%	63%	
Unresolved (%)	43%	37%	
Requiring follow-up treatment (%)	93.3%	36.7%	61%
Medication & treatment cost per OP appointment (£)	£199	£149	25%
Sutureless Omnigen cost - per application (£)	–	£400	
Sutureless Omnigen Applications - PP	–	1.20	–
Initial OP appointment cost (£)	£134	£134	
Subsequent OP appointment cost (£)	£59	£59	
Follow-up treatment cost (£pp)	£2411	£2411	
**OP Cost Initial Outcome (£pp)**			
Total OP medication & treatment cost	£1985	£1076	48%
Total OP appointments	£665	£547	19%
Total OP cost to the initial outcome	£2650	£1623	41%
**Addition Interventions (Follow-Up Treatments) (£pp)**			
Average Follow-up cost	£2249	£885	61%
**Total Average PP Cost**	£4900	£2508	50%
**Total Costs per 30 Patient Cohort**			
Treated Population (*n*)	30	30	
Total cost to initial outcome—Full Cohort (£)	£79,512	£48,690	41%
Total follow-up cost—Full Cohort (£)	£67,484	£26,551	61%
Total cost to end of follow-up—Full Cohort	£146,996	£75,241	50%

Based on published HAMD data, the average treatment course included 1.2 applications over an average of 98 days, with a total of eight OP visits (four associated with treatment, and four follow-up). The cost of HAMDM and lens (Omnigen £235; OmniLenz £120; postage £45) totalled £400 per application. Medication costs were adjusted from £199 per visit in SOC (Table 2) to £149 in the HAMDM group, reflecting replacement of adjunctive therapies like AMT and Prokera. Because all patients across both SoC and HAMDM cohorts were treated within the same NHS institutions, by the same clinical teams, and according to shared clinical pathways and treatment escalation protocols, a standardised per-patient follow-up treatment cost of £2249, based on the SOC data, was applied to HAMDM to allow direct economic comparison. As such, the nature and frequency of follow-up interventions were expected to be broadly comparable.

**Table 2:** Summary of OP visits, follow-up interventions, and total cost per patient by clinical aetiology of PED.

Indication	Patients *n* (%)	Mean OP Appointments (± SD) and Days Between	Prescribed Treatment Cost per OP Visit (£, ± SD)	Total OP Treatment Cost (£, ± SD) (Cost per Treatment Day)	* Follow-up Intervention Cost (£) (*n* = 30)	Overall Final Cost per-Patient (£) (*n* = 30)	Fold Increase in Total Cost
**Primary diagnosis (overall)**							
**PCED**	30 (100)	10.1 ± 4.4 (17.3)	199 ± 186	2650 ± 1773 (15.1)	2249 ± 2136	4900 ± 3363	1.9
**Secondary diagnosis**							
**LSCD, related to**	**11 (37)**	**10** ± **4 (24.5)**	**299** ± **195**	**3636** ± **1968 (14.8)**	**3712** ± **2626**	**7348** ± **3861**	**2.0**
*Chemical injury*	6 (20)	11.5 ± 3 (24.4)	330 ± 174	4189 ± 1319 (15.0)	4219 ± 2428	8408 ± 2806	2.0
*Rosacea*	2 (7)	6 ± 3 (24.1)	307 ± 277	2662 ± 2696 (18.4)	3959 ± 5130	6621 ± 7826	2.5
*Aniridia*	1 (3)	11 (13.7)	534	6598 (43.7)	3636	10,234	1.6
*Radiation*	1 (3)	15 (27.9)	78	2130 (5.1)	325	2455	1.2
*SJS*	1 (3)	5 (31.6)	89	813 (5.1)	3636	4449	5.5
**Microbial keratitis**	**6 (20)**	**10** ± **5 (7.5)**	**217** ± **229**	**2650** ± **1743 (35.3)**	**2586** ± **1989**	**5235** ± **2678**	**2.0**
*Alone*	4 (13)	7 ± 2 (11.6)	252 ± 287	2245 ± 2070 (27.7)	1555 ± 1533	3801 ± 1896	1.7
*with neurotrophic cornea*	1 (3)	19 (2.4)	142	3889 (84.5)	4646	8535	2.2
*with DSAEK*	1 (3)	14 (5.6)	152	3027 (38.8)	4646	7673	2.5
Neurotrophic cornea	6 (20)	9 ± 3 (11.7)	139 ± 152	1672 ± 833 (16.0)	555 ± 791	2226 ± 926	1.3
**Corneal-Surgery,**	**5 (17)**	**10** ± **4 (15.9)**	**79** ± **36**	**1481** ± **668 (9.3)**	**1801** ± **1703**	**3283** ± **2065**	**2.2**
*PK*	3 (10)	10 ± 5 (13.7)	70 ± 38	1355 ± 666 (9.9)	2532 ± 1912	3887 ± 2491	2.9
*keratoplasty*	1 (3)	12 (23.3)	125	2278 (8.1)	1076	3354	1.5
*DSAEK*	1 (3)	8 (12.8)	65	1067 (10.5)	332	1399	1.3
**Other**	**2 (6)**	**14** ± **10 (25.0)**	**67** ± **41**	**1630** ± **666 (4.7)**	**1821** ± **2566**	**3451** ± **1901**	**2.1**
Thyroid eye disease	1 (3)	21 (6.7)	37	2100 (15.0)	7	2107	1.0
Trauma	1 (3)	7 (80.0)	96	1159 (2.1)	3636	4795	4.1

Based on 30 out of the 30 patients achieving an outcome. Definitions: Outpatient (OP); Prescribed Treatment Cost per OP Visit reflects the average cost of standard therapies assumed to be prescribed at each visit; Total OP Treatment Cost includes OP appointment and medication costs from presentation to resolution; Cost per Treatment Day is the average total OP cost divided by treatment duration; *Follow-up Intervention Cost*: Includes additional interventions (e.g. surgery, long-term therapy) after initial OP care - * excluding additional OP visits and OP treatment costs; *Overall Final Cost per Patient*: Total cost combining OP treatment and follow-up treatments; *Fold Increase in Total Cost*: Ratio of final total cost vs. OP cost alone, reflecting added financial burden from follow-up treatments.

This methodology provided a robust framework to evaluate real-world management of PED, quantify resource utilisation, and compare the clinical and economic impacts of conventional treatment versus HAMDM-based intervention in a UK NHS setting.

## Results

### Patient cohort and baseline characteristics

Thirty eyes, specifically with refractory PED, from 30 patients were identified and included in the study. Patients were treated at Queen Victoria Hospital, East Grinstead (*n* = 20, 67%) and Maidstone and Tunbridge Wells NHS Trust (*n* = 10, 33%) (Table 3). This geographical spread may reflect regional variations in the prevalence or management strategies of ocular surface conditions, although the study’s scope does not extend to a comparative analysis. The mean age was 67 ± 19 years (range: 19–92 years), and 57% (*n* = 17) were male. Laterality was nearly evenly distributed, with right eye involvement in 43% (13/30) and left eye in 57% (17/30).

**Table 3:** Patient demographics and clinical diagnosis.

Characteristic/Diagnosis	Value/Frequency (%)
**Total Patients**	**30**
**Age (mean ± SD)**	**67 ± 19**
**Sex (male/female)**	17 (57)/13 (43)
**Laterality-Right**	13 (43)
**Primary diagnosis (overall)**	
PCED	30 (100)
**Secondary diagnosis**	
**LSCD, related to**	**11 (37)**
*Chemical injury*	6 (20)
*Rosacea*	2 (7)
*Aniridia*	1 (3)
*Radiation*	1 (3)
*SJS*	1 (3)
**Post-surgery, related to**	**5 (16)**
*Corneal transplant*	1 (3)
*Penetrating keratoplasty (PK)*	3 (13)
*DSAEK*	1 (3)
Neurotrophic cornea	6 (20)
**Microbial keratitis**	**6 (20)**
*Alone*	4 (13)
*with neurotrophic cornea*	1 (3)
*with DSAEK*	1 (3)
Thyroid eye disease	1 (3)
Trauma	1 (3)
**Comorbidities**	
Hypertension	21 (70)
Diabetes mellitus (DM)	14 (47)
Auto-immune disease	3 (10)
Cardiovascular disease	2 (7)
System infection	2 (7)
Connective tissue disorder	2 (7)
Neurodegenerative disease	0 (0)
Other	10 (33)
**Concomitant medication**	
**Standard-of-care**	
Topical antibiotics	30 (100)
Topical lubricants	30 (100)
Topical steroids	30 (100)
**Additional treatments**	
Tarsorrhaphy	25 (83)
Bandage contact lenses	22 (73)
Anti-proteases	17 (57)
Topical immunosuppressants	15 (50)
Systemic immunosuppressants	9 (30)
Serum / plasma eye drops	8 (27)
Botulinum toxin ptosis (Botox)	8 (27)
Punctual plugs	3 (10)
Systemic steroids	3 (10)
Ocular surgery	2 (7)
Oral antibiotics	2 (7)
Analgesia	1 (3)

*DSAEK* Descemet’s Stripping Automated Endothelial Keratoplasty, Diagnoses reflect underlying aetiologies contributing to persistent epithelial defects; Comorbidities were recorded from medical records at initial presentation.

Systemic comorbidities were highly prevalent; 80% (24/30) of patients had at least one coexisting systemic condition. The most common were hypertension (70%), diabetes mellitus (47%), and autoimmune diseases (10%). Twenty-seven percent (*n* = 8) had two comorbidities, and 30% (*n* = 9) had three or more (Table 3).

### Aetiology of persistent corneal epithelial defects (PED)

All patients (*n* = 30) were diagnosed with PEDs. The most common underlying condition was limbal stem cell deficiency (LSCD) in 37% (*n* = 11), followed by microbial keratitis (20%), neurotrophic cornea (20%), and post-surgical epithelial breakdown (16%). Less frequent causes included thyroid eye disease and trauma (Table 3).

### Conventional treatment modalities

All patients were initially managed with standard topical therapy, including topical lubricants, antibiotics, and corticosteroids. Adjunctive interventions were common; 83% (*n* = 25) received tarsorrhaphy, and 73% (*n* = 22) were fitted with bandage contact lenses (BCL). Additional treatments included topical immunosuppressants (50%), systemic immunosuppression (30%), and serum/plasma eye drops (27%). Botox-induced ptosis, punctal plugs, and systemic corticosteroids were used in selected cases. Thirty-seven percent of patients required at least three additional treatments, whilst 50% received four or more (Table 3).

Seventeen out of 30 (57%) cases achieved a complete resolution of their PED, while 43% (13/30) were classified as unresolved. Among the unresolved cases, 27% (8/30) had partial resolution, one patient (3%) showed no change, and four (13%) experienced clinical deterioration. Complete resolution was most frequently achieved in patients with neurotrophic keratitis (NK, 83%) and microbial keratitis (67%) (Table 4).

**Table 4:** Follow-up Interventions and Outcomes by Treatment Type.

Follow-up treatments	Patients *n* = 30 (%)	Complete resolution *n* = 17 (%)	Unresolved/Partial resolution *n* = 8 (%)	Deteriorated *n* = 4 (%)
Corneal graft (PK, lamella graft)	14 (47)	7 (41)	4 (50)	2 (50)
**AMT**	**10 (33)**	**6 (35)**	**3 (38)**	**1 (25)**
*- Conventional surgical AMT*	4 (13)	2 (12)	1 (13)	1 (25)
*- Sutureless OP AMT (Omnigen-OmniLenz)*	6 (20)	4 (24)	2 (25)	0
**BCL**	3 (10)	2 (12)	1 (13)	0
**Continued condition follow-ups (stabilise)**	3 (10)	2 (12)	1 (13)	0
**Lower-lid surgery**	3 (10)	3 (18)	0	0
**Evisceration**	2 (7)	0	0	2 (50)
**Limbal stem cell transplant (SLET and other)**	2 (7)	2 (12)	0	0
**Avastin**	2 (7)	1 (6)	0	1 (25)
**Orbital floor triamcinolone**	1 (3)	1 (6)	0	0

*PK* Penetrating keratoplasty, *AMT* Amniotic membrane transplantation, *BCL* Bandage contact lens.

Table shows frequency of follow-up interventions and corresponding clinical outcomes. Data excludes one lost to follow-up.

The mean duration of treatment from initial presentation to outcome was 176 ± 188 days (range 15–959). The longest treatment durations were associated with trauma (560 days), limbal stem cell deficiency (LSCD, 245 ± 255 days) and corneal transplantation (280 days) (Table 5).

**Table 5:** Healing outcomes and treatment durations by aetiology.

Indication	Patients *n* (%)	Complete resolution *n* (%)	Unresolved - Partial resolution *n* (%)	No change *n* (%)	Deterioration *n* (%)	Treatment duration (days)
Primary diagnosis						
PCED	30 (100)	17 (57)	8 (27)	1 (3)	4 (13)	176 ± 188
Secondary diagnosis						
**LSCD, related to**	**11 (37)**	**6 (55)**	**2 (18)**	**0**	**3 (27*)***	**245** ± **255**
*Chemical injury*	6 (20)	3 (27)	1 (9)	0	2 (18)	280 ± 337
*Rosacea*	2 (7)	2 (18)	0	0	0	145 ± 93
*Aniridia*	1 (3)	1 (9)	0	0	0	151
*Radiation*	1 (3)	0	1 (9)	0	0	418
*SJS*	1 (3)	0	0	0	1 (9)	158
***Microbial keratitis***	**6 (20)**	**4 (67)**	**1 (17)**	**1 (17)**	**0**	**75** ± **68**
*Alone*	4 (13)	2 (33)	1 (17)	1 (17)	0	81 ± 86
*+ neurotrophic cornea*	1 (3)	1 (17)	0	0	0	46
+ *DSAEK*	1 (3)	1 (17)	0	0	0	78
Neurotrophic cornea	6 (20)	5 (83)	1 (17)	0	0	105 ± 67
**Corneal-Surgery,**	**5 (17)**	**1 (20)**	**3 (60)**	**0**	**1 (10)**	**159** ± **91**
*Penetrating keratoplasty*	3 (10)	1 (20)	2 (40)	0	0	137 ± 84
*keratoplasty*	1(3)	0	1 (20)	0	1 (10)	280
*DSAEK*	1 (3)	0	0	0	0	102
**Other,**	**2 (7)**	**1 (50)**	**1 (50)**	**0**	**0**	**350** ± **297**
Thyroid eye disease	1 (3)	1 (50)	0	0	0	140
Trauma	1 (3)	0	1 (50)	0	0	560

### Outpatient (OP) resource use

Patients required an average of 10 ± 5 OP appointments (range 4–21), with an inter-visit interval of 17.3 days. Longer intervals between visits were noted in cases of LSCD and trauma (>21 days), whilst cases of microbial keratitis, neurotrophic cornea, and thyroid disease were managed with more frequent appointments (<14 days between appointments). Treatment costs and inter-visit patterns by aetiology are summarised in Table 2.

### Follow-up inventions and complications

At the conclusion of the audit period, one patient was fully healed and discharged without requiring additional follow-up interventions. Two out of 30 patients were lost to follow-up, one due to death during the audit period but received additional follow-up treatments, and one due to non-attendance at follow-up appointments. The latter was classified as healed at last review and was accounted as zero cost in the follow-up treatments. Ninety-three percent (28/30) required further follow-up interventions beyond initial management.

Common complications included anterior chamber inflammation (63%: *n* = 11, resolved; *n* = 8 unresolved), corneal neovascularisation (53%: *n* = 9, resolved; *n* = 7, unresolved), and corneal haze formation (53%: *n* = 10, resolved; *n* = 6, unresolved), with many (97%) patients exhibiting multiple findings.

The most frequently performed follow-up procedures were corneal grafts (48%), and amniotic membrane transplantation (AMT, 35%), a quarter of amniotic membrane transplantation (AMT) procedures using sutureless human amniotic membrane-derived dehydrated matrix (HAMDM, Omnigen, NuVision Biotherapies) in an OP setting (Table 4).

### Cost of conventual treatment (SOC)

The mean per patient cost of OP care, including consultations and prescribed medications, was £2650 ± £1773 (range £725-£6598). Total OP management cost across the 30-patient cohort was £79,512. When follow-up interventions were included for all 30 patients, the mean total cost per patient rose by £2249 ± £2136 (range £6.50-£8481) to £4900 ± £3363 (range: £864–£12,423) with an overall cohort cost of £146,996. SJS and ocular trauma-related cases incurred the highest per-patient costs, at 5.5-fold and 4.1-fold above the cohort mean, respectively (Table 2).

### Cost of HAMDM compared to SOC

We previously published two studies of patients presenting at Queen Victoria Hospital (QVH) and Maidstone and Tunbridge Wells Hospital (MTW) with ocular surface disease treated with a sutureless HAMDM [1, 22]. Pooled data from these studies (*n* = 127) were used for comparison. In these studies, patients received an average of 1.2 HAMDM applications, with a mean treatment time of 23 days and 8 OP visits over a 98-day period. Complete resolution was achieved in 63%, and partial resolution in 27%. Compared with conventional modalities in this study, the average treatment time was shorter, and fewer patients required further interventions (Table 1).

When modelled using the same unit cost assumptions, the average OP treatment cost per HAMDM patient was £1623 to outcome and £2508 including follow-up intervention care, representing a potential saving of £2392 per patient compared to SOC. Extrapolating to a 30-patient cohort, this would represent a cost saving of £71,755 (Table 1). The reduction in follow-up interventions and shorter treatment duration were the main determinants of differences in cost.

## Discussion

This retrospective study of patients managed for persistent corneal epithelial defect (PED) across two NHS centres provides valuable insights into the clinical and economic burden of a condition that remains difficult to manage and frequently recalcitrant to standard care within the NHS. The findings highlight significant variability in PED aetiology, the limitations of conventional treatment approaches, and the high resource utilisation associated with standard-of-care (SOC) management. Additionally, the comparison of SOC with human amniotic membrane-derived dehydrated matrix (HAMDM) underscores the potential to improve clinical outcomes while reducing healthcare costs.

### Clinical complexity and management challenges

Despite increased recognition of PED, its true incidence and economic implications remain poorly quantified. In the US, an estimated 200,000 cases occur annually, a figure likely underrepresenting the global burden [6, 23]. Applying these estimates to the UK yields an estimated 14,800 to 20,000 cases annually [6, 7], though true incidence may be higher due to non-specific NHS coding. Considering high-risk aetiologies such as microbial keratitis, corneal ulcers, diabetic keratopathy, penetrating keratoplasty (PK), ocular graft versus host disease (OGVHD), Neurotrophic Keratitis (NK), chemical eye injuries (CEI), and LSCD [2], the UK PED incidence may reach 38,000 cases annually [24–30].

PED aetiology in this cohort was diverse, with the most common diagnoses being LSCD, microbial keratitis, NK, and post-surgical epithelial breakdown. These are consistent with previously reported causes, including epithelial adhesion anomalies (e.g. recurrent corneal erosions), tear disorders (e.g. dry eye disease, trachoma, Sjögren’s), and other systemic conditions, which underscores the multifactorial pathophysiology of PED [8, 23]. While comorbidities did not independently predict outcome, they likely contributed to delayed healing, recurrent disease, and therapeutic complexity [6, 8, 23, 31–35]. In support of this, our study revealed a high prevalence of systemic comorbidities within the cohort, with hypertension affecting 70% of patients and diabetes mellitus present in 47% (Table 3). These chronic conditions are well-documented risk factors for impaired corneal epithelial healing and have been associated with poorer outcomes in ocular surface disease, including PED with diabetes, in particular, contributes to neurotrophic dysfunction and tear film instability, which can exacerbate epithelial breakdown [23, 32, 34, 35]. The presence of such comorbidities may therefore serve as early indicators of refractory disease and justify earlier intervention with HAMDM. Stratifying patients based on systemic risk factors may enable more proactive treatment planning, potentially improving outcomes and reducing prolonged NHS resource utilisation in this costly cohort.

Despite the use of standard treatments, including lubricants, antibiotics, corticosteroids, and adjunctive interventions such as tarsorrhaphy (83%) and BCL (73%), only 57% of patients achieved complete healing. A substantial proportion of the cohort required continued follow-up/surgical interventions, reflecting the limitations of conventional methodologies in refractory PED. The prolonged average treatment duration of 176 days further demonstrates the inefficiencies in resource utilisation and care delivery [35, 36]. Outcome variability was also noted by aetiology: LSCD and post-corneal surgery cases demonstrated lower healing rates compared to infectious and neurotrophic causes. Additionally, the high rate of follow-up interventions in the cohort, including corneal grafting (47%) and AMT (37%), suggests that SOC alone may not be sufficient for refractory PED.

The cost analysis highlights the substantial costs of the current management of PED. The average per-patient cost was £4900 when follow-up interventions were included, and the total cost for managing the 30-patient cohort was more than £146,000. Previous studies reinforce these findings: microbial keratitis, accounting for 20% of this cohort, is a leading cause of corneal blindness and incurs high direct costs in both the US and UK [18, 19]. Similar economic patterns are observed for NK in other European health systems [28].

### Management strategies

PED offers a therapeutic window wherein timely intervention can prevent progression to corneal opacity or vision loss, unlike incurable degenerative diseases such as glaucoma [37]. However, managing PED requires a balance of interventions to prevent severe complications such as infection, ulceration, and corneal perforation [23]. This study highlights the central role of conventional treatments, including lubricants, antibiotics, and corticosteroids, in initial management [23, 38]. However, the high frequency need for additional interventions such as tarsorrhaphy, BCL, serum eye drops (SED) and immunosuppressants reflects the chronicity and refractory nature of PED [4, 8, 38].

### Conventional PED treatment options

Among adjunctive treatments, BCLs SED are commonly used for persistent or recurrent cases. BCLs used for approximately 21 days have shown improved healing rates of up to 55% complete healing [8, 38–40]. In this audit, 60% of patients receiving both BCLs and tarsorrhaphy achieved a 50% healing rate. Although BCL use may increase infection risk via tear film stagnation [41, 42], this risk can be mitigated with concurrent antibiotic use [40, 43]. SED, applied alone or after BCL, provides lubrication and epitheliotropic support [8]. In this cohort, 25% of patients received SED, most in combination with or following BCL application, and 50% achieved complete resolution. Literature suggests higher resolution rates (83–100%) with prolonged SED use [10–12, 44–47]. However, recurrence remains common after discontinuation (up to 50%), and eligibility criteria, excluding patients with systemic inflammation or active infections, limit broader application [48, 49].

Emerging treatments such as epidermal growth factor (EGF), insulin-like growth factor (IGF), and nerve growth factor (NGF) demonstrate biological promise but face costs, accessibility, and efficacy hurdles limiting widespread adoption [15, 50–58].

#### AMT as a treatment for refractory PED

AMT is an established adjunct surgical therapy in PED, promoting epithelial repair and inflammation reduction. Resolution rates of up to 97% and visual improvement in over 50% of cases are reported from surgical application [5, 59]. In this retrospective cohort, 10 patients received AMT with a 60% success rate. Outcomes were most favourable in infectious and neurotrophic aetiologies, consistent with prior literature [5, 59]. However, conventional AMT requires operating room resources and incurs surgical costs, limiting early-stage accessibility. Reported failure rates for surgical AMT range from 10–30% [60–62].

Sutureless AMT represents a key innovation. It permits outpatient, non-surgical delivery, improving accessibility and reducing system costs. While traditional sutureless AMT reports lower resolution rates (~45.5%) [63], newer technologies such as HAMDM offer better outcomes with less complexity. Our previous study using sutureless HAMDM (Omnigen) reported a 63% resolution rate with just 1.2 applications and a mean treatment time of 25 days [1]. Only 14% of cases required further surgical intervention, and patients remained defect-free for at least 121 days [1]. These outcomes are supported by prospective studies showing near-complete resolution with early HAMDM use [64, 65], which, based on this study, would significantly reduce healthcare resource burden and associated costs. Ho et al. further demonstrated that earlier intervention with sutureless HAMDM reduces the necessity for additional interventions and follow-up demand [64].

#### HAMDM as an alternative

Pooling data from Maqsood et al.[1, 22]. enabled modelling of a cost comparison between SOC and sutureless HAMDM. HAMDM-treated patients achieved higher resolution rates (63% vs. 57%), required fewer overall OP visits (eight vs. ten), and showed shorter mean treatment duration (98 vs. 176 days). The average per-patient cost was reduced from £4900 (SOC) to £2508 (HAMDM), resulting in an estimated potential saving of £2.392 per patient. Shorter follow-up, fewer surgical interventions, and shorter healing time underpinned these savings [1, 64, 65]. The reduced treatment duration (98 vs. 176 days) translated into less resource utilisation and reduced clinical burden. The findings suggest that integrating HAMDM into the routine, and even earlier, in the treatment pathway could reduce PED treatment costs while leading to faster recovery and better clinical outcomes.

#### National statistics

To contextualise the clinical findings of this study, a review of NHS Digital Hospital Episode Statistics (HES) for the period April 2017 to April 2018 was performed [66–68]. The goal was to understand the national scope of corneal surface disease episodes, acknowledging that no specific ICD-10 code exists for PED. Relevant cases were identified using a range of corneal surface disease codes (H160–H193), including corneal ulcers and epithelial defects.

Data capture was inconsistent: only 8 of 170 ophthalmic A&E providers and 20 of 209 OP providers reported data, compared with 176 inpatient providers. These discrepancies underscore systemic issues in coding and data completeness, particularly in OP and emergency services. Despite this, the data revealed a large and under-recognised burden of chronic corneal epithelial pathology being managed in OP settings, with 161,471 corneal surface disease-related episodes across all care settings. These included 55,366 (34%) episodes in A&E, 96,181 (60%) in OP care, and 9924 (6%) in Admitted Patient Care (APC). Among these, 138,831 episodes were estimated to be related to corneal ulcers (H160), epithelial defects (H188), or PED (H189) (Supplementary Table 1). Notably, the majority of H188 and H189 episodes were seen in OP settings (93%), with far fewer in A&E (6%) and inpatient care (1%), while corneal ulcers were more likely to present in A&E (77%). This aligns with the chronic OP nature of PED management and highlights an under-recognised service burden (Supplementary Table 1). Although cost modelling was not attempted, the scale of OP care reinforces the potential impact of earlier, efficient treatments like HAMDM. These findings reinforce the need for improved recognition, coding, and management strategies for PED within the NHS.

### Study limitations

While this study provides valuable insights into PED management within the NHS, several limitations must be acknowledged. Its retrospective design and restriction to two NHS centres limit the generalisability of findings across broader care settings. Additionally, NHS coding limitations may have biased the identification of cases toward more severe or refractory PED, potentially underestimating the true spectrum and prevalence of the condition.

The HES data analysis provided a useful national context but must be interpreted with caution due to incomplete OP and A&E reporting, variations in coding practices, and the lack of a specific ICD-10 code for PED. These limitations hinder precise burden estimation and underscore the need for improved data capture across care settings.

The cost comparison between SoC and HAMDM-treated patients is limited because of the relatively small sample sizes, and because outcome and resource use data are specific to the hospitals and clinicians involved in the included studies and are not necessarily generalisable. However, the significant clinical benefits of HAMDM suggest that an adequately powered comparative study would produce similar results.

### Future direction of research

In light of the potentially important benefits of HAMDM, prospective comparative studies with larger, multicentre cohorts and integrated health-economic evaluation are warranted. Future studies should include the impact of treatment on patient quality of life and on efficiency gains within the healthcare system made possible by releasing resources for the treatment of additional patients.

## Conclusion

This study highlights the substantial clinical and economic burden of PED within the NHS and suggests that HAMDM is an important, clinically effective and economically viable alternative to SOC. Although conventional therapies remain integral to current management, the high proportion of unresolved cases and the frequent need for additional interventions emphasise the limitations of SOC in treating PED.

By comparison, patients treated with HAMDM demonstrated shorter treatment durations, fewer OP visits, and lower costs while achieving higher resolution rates. These findings suggest that broader adoption of innovative treatments such as HAMDM could improve clinical outcomes, enhance patient experience, and free up NHS resources. In the context of rising demand for ophthalmic services, integrating HAMDM earlier in the treatment pathway may offer a strategic opportunity to optimise PED care and improve health system efficiency.

## Summary

### What is known about this topic

- Despite increased recognition of PED, its true incidence and economic implications remain poorly quantified.
- Chronic PEDs, though relatively infrequent in incidence, generate high cumulative burden due to prolonged healing time, frequent monitoring, and the need for surgical rescue therapy.

### What this study adds

- This study highlights the substantial clinical and economic burden of PED within the NHS.
- Patients treated with HAMDM demonstrated shorter treatment durations, fewer Outpatient visits, and lower costs while achieving higher resolution rates.

## Supplementary information

Supplementary Table 1

## Data Availability

The datasets generated during and/or analysed during this study are available from the corresponding author on reasonable request.
