# Supplementary material for: Management of ocular surface disease involving inflammation and persistent epithelial defects utilising various treatment modalities in the UK National Health Service (NHS)
Source: Eye (Lond). 2026 Jun 4;40(11):1627–36. doi: 10.1038/s41433-026-04314-6 (PMC13415519; doi:10.1038/s41433-026-04314-6)
Supplement: Supplementary file 1 — Supplementary Table 1 [file 41433_2026_4314_MOESM1_ESM.docx]

Supplementary Table 1. Estimated Hospital Episodes for Corneal Ulcers and Epithelial Defects (H160, H188, H189)

| Setting | **Corneal Ulcer (H160)** | | **Epithelial defects (H188)** | | **PED**  **(H189)** | | **TOTAL** |
| --- | --- | --- | --- | --- | --- | --- | --- |
| A&E | 46,624 | 79% | 8,742 | 15% | 3,885 | 7% | **59,252** |
|  | **77%** |  | **62%** |  | 6% |  |  |
| OP | 12,023 | 16% | 4,372 | 6% | 60,113 | 79% | **76,508** |
|  | **20%** |  | 31% |  | **93%** |  |  |
| APC | 1,654 | 54% | 1,063 | 35% | 354 | 12% | **3,072** |
|  | 3% |  | 7% |  | 1% |  |  |
| **TOTAL** | **60,301** | 43% | **14,177** | 10% | **64,353** | 46% | **138,831** |
